# Serum uric acid variability and short-term mortality in chronic kidney disease: a retrospective cohort study with independent replication in MIMIC-IV

**DOI:** 10.3389/fnut.2026.1919779

**Published:** 2026-09-16

**Authors:** Fan Zhu, Wenyuan Gan, Wenli Chen, Xingruo Zeng

**Affiliations:** 1 Department of Nephrology, The Central Hospital of Wuhan, Tongji Medical College, Huazhong University of Science and Technology, Wuhan, Hubei, China; 2 Key Laboratory for Molecular Diagnosis of Hubei Province, The Central Hospital of Wuhan, Tongji Medical College, Huazhong University of Science and Technology, Wuhan, Hubei, China; 3 Department of Nutrition, The Central Hospital of Wuhan, Tongji Medical College, Huazhong University of Science and Technology, Wuhan, Hubei, China

**Keywords:** biological variability, chronic kidney disease, electronic health records, MIMIC-IV, serum uric acid, short-term mortality

## Abstract

**Inroduction:**

Within-person variation in serum uric acid (SUA) may reflect clinical instability, but its association with outcomes in chronic kidney disease (CKD) is uncertain.

**Methods:**

We studied 10,556 adults with EHR-defined CKD and at least three SUA measurements during a 365-day baseline window across a nine-hospital consortium. Variability was quantified primarily by the coefficient of variation (CV); the primary outcome was all-cause mortality, and secondary outcomes were a composite kidney endpoint and major adverse cardiovascular events (MACE). Cox models adjusted for demographics, comorbidities, baseline laboratory values, mean SUA, measurement number and medication use. Reproducibility of the short-term mortality association was assessed in MIMIC-IV.

**Results:**

During a median follow-up of 0.62 years, 649 deaths, 676 kidney events and 3,172 first MACE occurred. Higher SUA CV was associated with mortality in the fully adjusted model (hazard ratio [HR] per 1-SD increase 1.24, 95% CI 1.15–1.32; highest versus lowest tertile 1.87, 95% CI 1.50–2.33), whereas adjusted associations with kidney events (HR 1.01, 95% CI 0.93–1.10) and MACE (HR 1.02, 95% CI 0.98–1.05) were null. Of all deaths, 495 (76.3%) occurred within 90 days. In a post-90-day landmark analysis of 6,784 participants with more than 90 days of follow-up, the mortality association was not observed (154 deaths; HR 1.02, 95% CI 0.86–1.21). Results were similar after daily aggregation of SUA measurements and additional adjustment for urate-lowering therapy, colchicine and sodium-glucose cotransporter-2 inhibitors. In MIMIC-IV (*n* = 5,350; 931 deaths within 90 days), the adjusted HR per 1-SD increase in CV was 1.32 (95% CI 1.26–1.38).

**Discussion:**

Greater SUA variability was associated with mortality during short follow-up; the concentration of deaths within 90 days and the null post-90-day landmark estimate suggest that the exposure may primarily mark near-term clinical deterioration rather than a causal or durable long-term prognostic mechanism.

## Introduction

Chronic kidney disease (CKD) affects more than 10% of adults worldwide and is a major cause of premature death, most often from cardiovascular causes (1). Serum uric acid (SUA), the terminal product of dietary and endogenous purine metabolism, is associated with hypertension, endothelial dysfunction, oxidative stress, cardiovascular events and death. Meta-analyses involving more than 1 million participants show a graded association with cardiovascular mortality, and kidney function modifies the relationship between SUA and outcomes (2–7).

Beyond average concentrations, within-person biomarker variability may capture instability in physiology or care. Variability in blood pressure, glycated haemoglobin, lipids and estimated glomerular filtration rate (eGFR) has been associated with cardiovascular and kidney outcomes (8–12). SUA varies with kidney function, volume status, diet, alcohol intake, medications, inflammation and intercurrent illness (13). Routine-care EHR data increasingly support longitudinal biomarker research and acute kidney injury phenotyping, while acute illness and nephrotoxic exposures can complicate interpretation of laboratory trajectories (14–16). Accordingly, variability in serial SUA measurements could reflect either a durable risk phenotype or acute clinical deterioration. Whether serial SUA variability is associated with outcomes in CKD, and over what time horizon, remains uncertain.

We therefore examined associations of serial SUA variability with all-cause mortality, kidney disease progression and major adverse cardiovascular events (MACE) in adults with EHR-defined CKD. We also characterised measurement timing and missingness, evaluated medication-related and outcome-definition sensitivities, assessed whether the mortality association persisted beyond 90 days, and tested reproducibility of the short-term signal in the MIMIC-IV critical-care database. Given the observational design and the short follow-up, our objective was to estimate associations rather than causal effects or long-term predictive performance.

## Materials and methods

### Study design and population

We conducted a retrospective cohort study using structured electronic health records from the hospital consortium led by The Central Hospital of Wuhan, an alliance of nine institutions (The Central Hospital of Wuhan and eight partner hospitals) in Wuhan, China. Adults aged at least 18 years were eligible if they had EHR-defined CKD and at least three SUA measurements during the 365 days before and including the index date; the last SUA measurement in that window defined the index date. CKD was operationalised as eGFR <90 mL/min/1.73 m^2^ with evidence of kidney damage when available, eGFR <60 mL/min/1.73 m^2^ on at least two occasions, or a structured record of CKD or chronic renal failure. Structured diagnoses from inpatient or outpatient encounters were accepted. Albuminuria or proteinuria contributed evidence of kidney damage when available but was not required for patients qualifying by a recorded CKD diagnosis. A uniform interval of at least 3 months was not imposed across all pathways, and a recorded diagnosis was not independently re-confirmed; an acute kidney injury code alone did not establish CKD. Thus, some misclassification from transient kidney dysfunction or diagnostic coding remains possible. Of 11,578 potentially eligible patients, 1,011 were excluded for incomplete follow-up or missing index information and 11 for age <18 years, leaving 10,556 participants (Figure 1). Reporting follows the STROBE statement (17).

**Figure 1:**
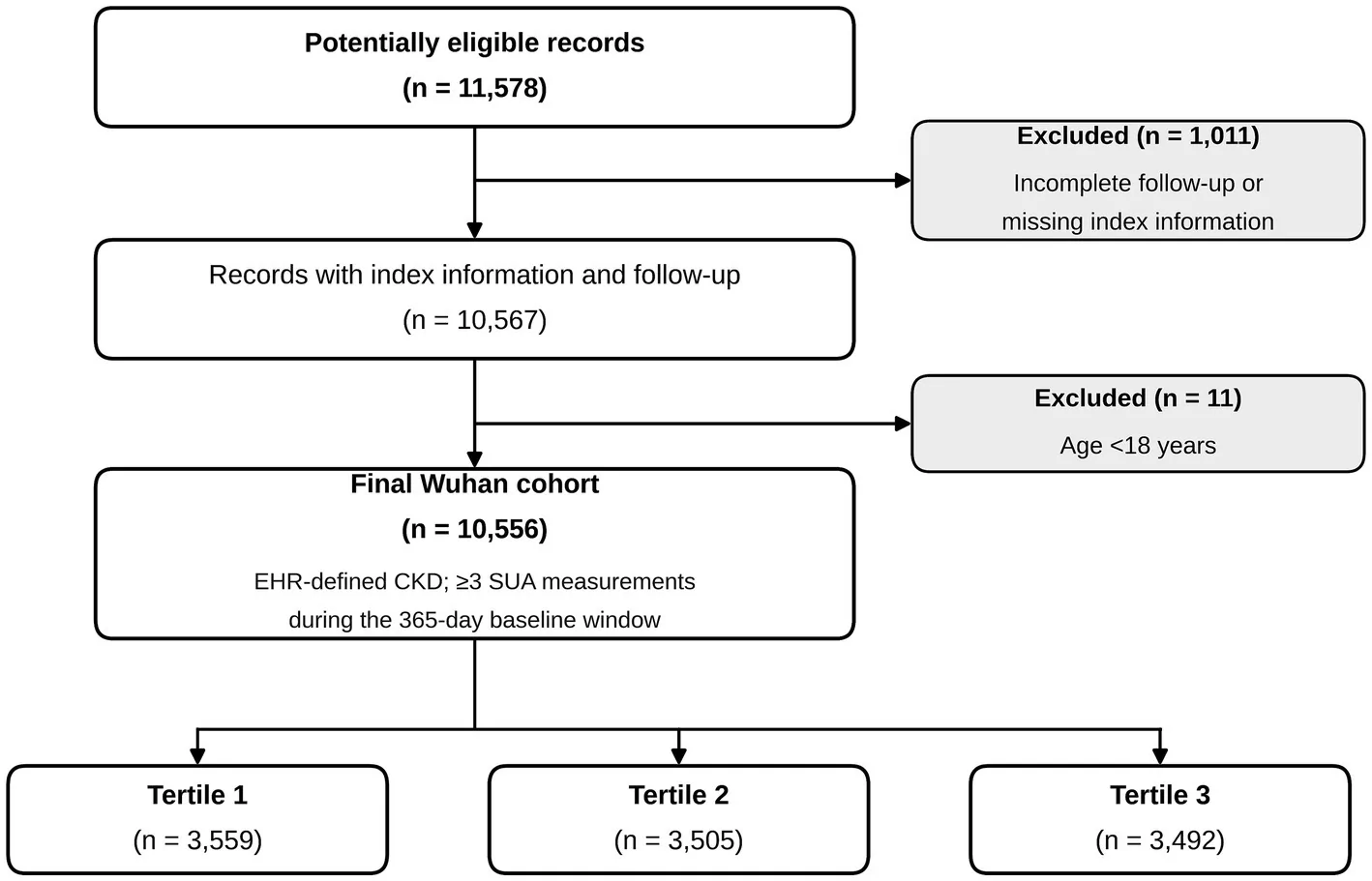
Study flow diagram. The derivation cohort comprised adults with EHR-defined CKD and at least three SUA measurements during the 365-day baseline window.

### SUA exposure and measurement timing

All available SUA results within the 365-day baseline window were used, irrespective of recorded care setting because outpatient versus inpatient provenance was not reliably available in the extract. The primary variability measure was CV (100 × standard deviation/mean). Average successive variability (ASV) and variability independent of the mean (VIM) were alternative measures. Participants were grouped into CV tertiles, with the lowest tertile as reference. Mean SUA and the number of measurements were retained as covariates. We summarised measurement number, elapsed time between the first and last SUA values and the within-patient interval between successive distinct measurement dates. Because repeated values could occur on the same calendar day, a sensitivity analysis first averaged all same-day values and then required at least three distinct dates before recalculating CV.

### Outcomes and ascertainment

The primary outcome was all-cause mortality, ascertained from inpatient records, the hospital death registry and linked cause-of-death records. The secondary kidney outcome was a composite of kidney failure (maintenance dialysis, kidney transplantation or a recorded eGFR <15 mL/min/1.73 m^2^) or the first observed decrease of at least 40% in eGFR from baseline. The main kidney definition did not require confirmation and could therefore include transient acute kidney injury. Sensitivity analyses required a subsequent eGFR value remaining at least 40% below baseline after a minimum of 28 or 90 days. The cardiovascular outcome was the first post-index MACE, defined as non-fatal myocardial infarction, non-fatal stroke, hospitalisation for heart failure or cardiovascular death; recurrent events were not counted. Outcomes were derived algorithmically from structured diagnoses, hospitalisation and linked event records without blinded manual adjudication. Follow-up extended from the index date to the first relevant event, death or last recorded contact, as appropriate.

### Covariates and medication exposure

Covariates included age, sex, diabetes, hypertension, coronary heart disease, heart failure, recorded hyperuricaemia diagnosis, prior stroke, chronic obstructive pulmonary disease, hyperlipidaemia, baseline eGFR calculated with the 2021 CKD-EPI creatinine equation (18), albumin, haemoglobin, glucose, mean SUA, number of SUA measurements, and use of statins, diuretics, non-steroidal anti-inflammatory drugs (NSAIDs) and renin-angiotensin-aldosterone system (RAAS) inhibitors. Hyperuricaemia was a diagnosis-based variable rather than a biochemical threshold; medication users were not excluded. Medication exposure was defined from prescriptions overlapping the 365-day baseline window. In sensitivity analyses, we additionally adjusted for urate-lowering therapy (allopurinol, febuxostat, benzbromarone or other uricosuric therapy), colchicine and sodium-glucose cotransporter-2 (SGLT2) inhibitors.

### Missing data

Baseline eGFR and mean SUA were complete. Albumin was missing for 402 participants (3.8%), haemoglobin for 1,014 (9.6%) and glucose for 1 (0.01%). Because missingness was limited, missing continuous covariates were replaced with the cohort median in the primary model. A complete-case analysis excluding all participants with missing adjustment covariates was performed as a sensitivity analysis.

### Statistical analysis

Continuous variables are reported as mean (standard deviation [SD]) or median (interquartile range [IQR]) and categorical variables as number (percentage). Three Cox models were fitted for each outcome: Model 1 was unadjusted; Model 2 adjusted for age and sex; Model 3 included all covariates listed above. CV was analysed per 1-SD increase and by tertile; linear trend was tested with the tertile ordinal term. Restricted cubic splines with five knots examined non-linearity. Cumulative incidence curves used Kaplan–Meier estimates with log-rank tests. For display, these curves were limited to 2 years because risk sets were sparse thereafter; all Cox models retained the full available follow-up. Prespecified subgroup models included interaction terms. Proportional hazards were assessed globally and for individual covariates using Schoenfeld-residual diagnostics; an exposure-by-log(time) interaction provided an additional exposure-specific test. To address reverse causation, a post-90-day landmark analysis retained participants with more than 90 days of follow-up, reset the time origin to day 90 and evaluated subsequent mortality. Further sensitivity analyses used ASV, VIM, SUA SD, winsorised CV, complete cases, additional blood-pressure adjustment, stricter measurement-number criteria, daily aggregation, medication adjustment and alternative confirmation requirements for the kidney outcome. Cox models used the Efron approximation for ties. Tests were two-sided and *p* < 0.05 was considered statistically significant.

### Independent replication in MIMIC-IV

We used MIMIC-IV version 3.1, a publicly available, de-identified EHR database from a United States academic medical centre (19). Adults with at least three SUA measurements were included; the last measurement defined the index date and the outcome was all-cause mortality within 90 days. Models adjusted for age, sex, mean SUA, number of measurements and eGFR. This analysis was designed to assess reproducibility in a critically ill population, not transportability to another ambulatory CKD cohort. Because CV is dimensionless, estimates could be compared despite different SUA assay units.

### Ethics

The study was conducted in accordance with the Declaration of Helsinki. The study protocol was approved by the institutional Ethics Committee of Wuhan Central Hospital (Approval No. WHZXKYL2022-112). The requirement for individual informed consent was waived owing to the retrospective use of de-identified data. This approval and consent waiver covered all nine institutions participating in the regional 1 + 8 medical consortium. The MIMIC-IV analysis used a publicly available de-identified database; the investigators completed the required PhysioNet credentialing and data-use agreement.

## Results

### Cohort characteristics and SUA measurement timing

The 10,556 participants had a mean age of 67.7 years (SD 13.9), 56.8% were men, 50.0% had diabetes and 78.9% had hypertension. Recorded hyperuricaemia was present in 45.3% (Table 1). Mean baseline eGFR was 71.8 mL/min/1.73 m^2^ (SD 27.0), and 3,678 participants (34.8%) had eGFR <60 mL/min/1.73 m^2^. UACR was available during baseline for 3,809 participants (36.1%). The median number of SUA measurements was 5 (IQR 3–8; mean 6.56, SD 5.55; range 3–100). The median span from the first to last SUA measurement was 231 days (IQR 68–319), and the median within-patient interval between successive distinct measurement dates was 26 days (IQR 4–76). Same-day duplicate measurements occurred in 2,583 participants (24.5%). Patients in the highest CV tertile were older, underwent more SUA measurements, and more often had heart failure, recorded hyperuricaemia and chronic obstructive pulmonary disease; they also had lower albumin and haemoglobin (Table 1).

**Table 1:** Baseline characteristics by tertile of serum uric acid coefficient of variation (CV).

Characteristic	Overall (*n* = 10,556)	T1 (*n* = 3,559)	T2 (*n* = 3,505)	T3 (*n* = 3,492)	*p*
Age, years	67.7 (13.9)	67.2 (13.7)	66.8 (14.1)	69.1 (13.8)	<0.001
Mean SUA, μmol/L	383.5 (107.0)	388.9 (106.8)	384.2 (103.5)	377.4 (110.3)	<0.001
SUA CV, %	20.8 (12.1)	9.3 (3.2)	18.8 (2.8)	34.5 (9.8)	<0.001
No. of SUA measurements	6.6 (5.5)	4.7 (2.8)	6.8 (5.1)	8.2 (7.3)	<0.001
eGFR, mL/min/1.73 m^2^	71.8 (27.0)	72.0 (26.8)	72.5 (27.0)	70.7 (27.1)	0.016
Albumin, g/L	38.6 (6.5)	39.6 (6.0)	39.0 (6.3)	37.3 (6.9)	<0.001
Haemoglobin, g/L	120.4 (24.1)	123.6 (23.3)	121.6 (23.6)	116.0 (24.8)	<0.001
Glucose, mmol/L	7.0 (3.2)	6.9 (3.1)	7.0 (3.2)	7.1 (3.3)	0.182
Systolic BP, mmHg	127.7 (19.9)	129.6 (20.1)	127.9 (20.2)	125.8 (19.2)	<0.001
Diastolic BP, mmHg	74.3 (12.3)	75.3 (12.2)	75.0 (12.3)	72.9 (12.3)	<0.001
Male sex	6,000 (56.8)	2,076 (58.3)	1,969 (56.2)	1,955 (56.0)	0.087
Diabetes	5,280 (50.0)	1,802 (50.6)	1,746 (49.8)	1,732 (49.6)	0.657
Hypertension	8,329 (78.9)	2,833 (79.6)	2,763 (78.8)	2,733 (78.3)	0.385
Coronary heart disease	6,292 (59.6)	2,102 (59.1)	2,080 (59.3)	2,110 (60.4)	0.470
Heart failure	4,034 (38.2)	1,246 (35.0)	1,316 (37.5)	1,472 (42.2)	<0.001
Recorded hyperuricaemia diagnosis	4,784 (45.3)	1,326 (37.3)	1,558 (44.5)	1,900 (54.4)	<0.001
Prior stroke	4,871 (46.1)	1,669 (46.9)	1,543 (44.0)	1,659 (47.5)	0.008
COPD	4,457 (42.2)	1,405 (39.5)	1,458 (41.6)	1,594 (45.6)	<0.001
Hyperlipidaemia	5,602 (53.1)	2,003 (56.3)	1,849 (52.8)	1,750 (50.1)	<0.001
Statin	6,980 (66.1)	2,381 (66.9)	2,286 (65.2)	2,313 (66.2)	0.324
Diuretic	5,771 (54.7)	1,635 (45.9)	1,914 (54.6)	2,222 (63.6)	<0.001
NSAID	7,523 (71.3)	2,437 (68.5)	2,488 (71.0)	2,598 (74.4)	<0.001
RAAS inhibitor	5,104 (48.4)	1,691 (47.5)	1,709 (48.8)	1,704 (48.8)	0.470

Data are mean (SD) or *n* (%). Hyperuricaemia denotes a recorded diagnosis rather than a sex-specific biochemical threshold; medication users were included. BP, blood pressure; COPD, chronic obstructive pulmonary disease; eGFR, estimated glomerular filtration rate; NSAID, non-steroidal anti-inflammatory drug; RAAS, renin-angiotensin-aldosterone system; SUA, serum uric acid.

### SUA variability and all-cause mortality

During a median follow-up of 0.62 years (IQR 0.04–1.19; 7,304 person-years), 649 deaths occurred: 121, 188 and 340 in CV tertiles 1, 2 and 3, respectively. The unadjusted curves, displayed through 2 years, separated across tertiles (log-rank *p* < 0.001; Figure 2A). In Model 3, the HR was 1.24 (95% CI 1.15–1.32; *p* < 0.001) per 1-SD increase in CV and 1.87 (95% CI 1.50–2.33) for the highest versus lowest tertile (*p* for trend <0.001; Table 2). Alternative metrics produced similar estimates (ASV HR 1.14, 95% CI 1.05–1.23; VIM HR 1.23, 95% CI 1.15–1.31). The restricted cubic spline indicated non-linearity, with risk increasing mainly above the median CV (*p* for non-linearity = 0.011; Figure 3). The global Schoenfeld-residual test indicated non-proportionality in the full model (*p* < 0.001), whereas the exposure-specific CV-by-log(time) interaction was not significant (*p* = 0.563).

**Figure 2:**
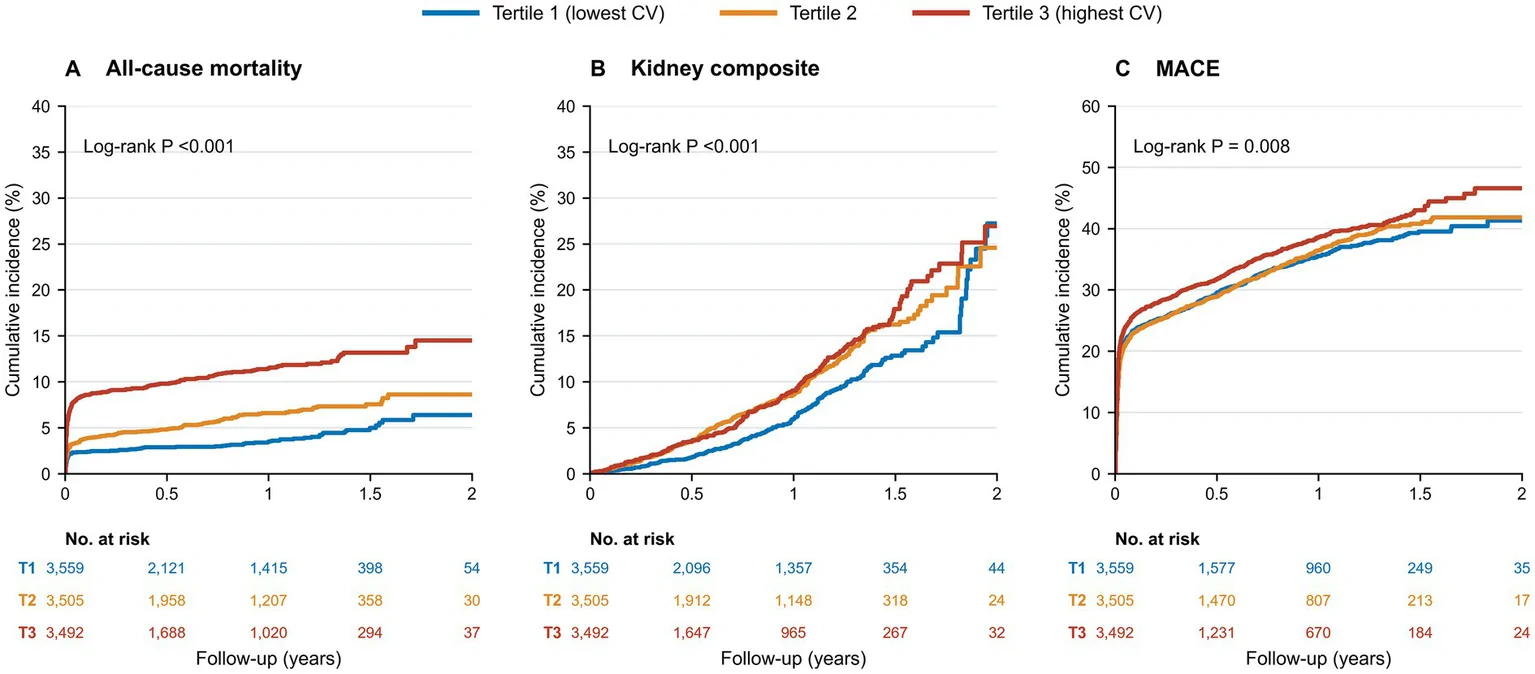
Unadjusted Kaplan–Meier cumulative incidence curves by tertile of serum uric acid CV. **(A)** All-cause mortality; **(B)** Composite kidney endpoint; and **(C)** MACE. Curves are displayed from 0 to 2 years because only 17–54 participants per tertile remained at risk at 2 years across the three outcomes; Cox models used all available follow-up. *p* values are from log-rank tests. The significant unadjusted separation for kidney events and MACE should be interpreted alongside the fully adjusted null estimates in Table 4.

**Table 2:** Hazard ratios for all-cause mortality by serum uric acid variability.

SUA variability	Model 1	Model 2	Model 3 (full)
Per 1-SD increase in CV	1.58 (1.49–1.68)	1.52 (1.44–1.62)	1.24 (1.15–1.32)
Tertile 1	1.00	1.00	1.00
Tertile 2	1.65 (1.31–2.07)	1.70 (1.35–2.14)	1.41 (1.12–1.78)
Tertile 3	3.22 (2.62–3.96)	2.99 (2.43–3.68)	1.87 (1.50–2.33)
*p* for trend	<0.001	<0.001	<0.001

Values are HR (95% CI). Model 1 was unadjusted. Model 2 adjusted for age and sex. Model 3 additionally adjusted for diabetes, hypertension, coronary heart disease, heart failure, recorded hyperuricaemia diagnosis, prior stroke, chronic obstructive pulmonary disease, hyperlipidaemia, baseline eGFR, albumin, haemoglobin, glucose, mean SUA, number of measurements, statins, diuretics, NSAIDs and RAAS inhibitors. Alternative metrics in Model 3: VIM 1.23 (1.15–1.31); ASV 1.14 (1.05–1.23). Cox models used all available follow-up; the 2-year limit applies only to the graphical display in Figure 2. CI, confidence interval; CV, coefficient of variation; HR, hazard ratio; SD, standard deviation; VIM, variability independent of the mean.

**Figure 3:**
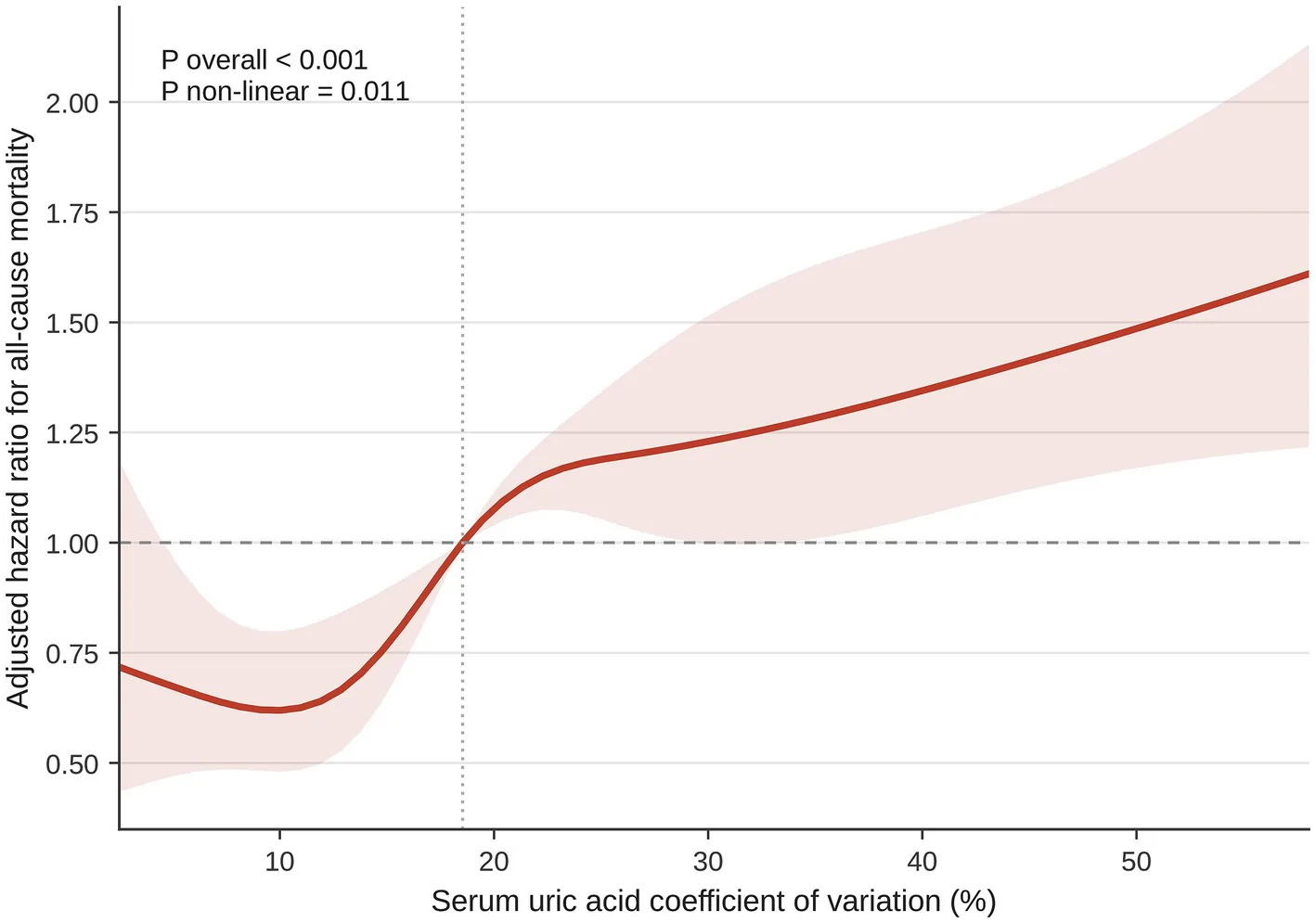
Restricted cubic spline of the fully adjusted hazard ratio for all-cause mortality across continuous serum uric acid CV, with the median as reference. The shaded band denotes the 95% confidence interval; *p* for non-linearity = 0.011.

### Timing of mortality and reverse-causation analysis

Deaths occurred a median of 4 days after the index date (IQR 2–75); 382 (58.9%) occurred within 7 days, 461 (71.0%) within 30 days and 495 (76.3%) within 90 days. In the post-90-day landmark analysis, 154 deaths occurred among 6,784 participants with more than 90 days of follow-up and the association was not observed (HR per 1-SD CV 1.02, 95% CI 0.86–1.21; *p* = 0.807; Table 3). The confidence interval does not exclude a modest association, but the pronounced temporal concentration and null landmark estimate are compatible with reverse causation and suggest that SUA variability may primarily mark near-term clinical deterioration.

**Table 3:** All-cause mortality per 1-SD CV across special populations and sensitivity analyses.

Population/analysis	Events/*n*	HR (95% CI)	*p*
Primary analysis			
Overall	649/10,556	1.24 (1.15–1.32)	<0.001
Special populations			
Diabetes mellitus	278/5,280	1.26 (1.12–1.41)	<0.001
Coronary heart disease	388/6,292	1.30 (1.18–1.43)	<0.001
Heart failure	345/4,034	1.24 (1.12–1.38)	<0.001
CKD G3a-G5 (eGFR <60)	383/3,678	1.34 (1.22–1.47)	<0.001
Age ≥75 years	362/3,267	1.21 (1.10–1.34)	<0.001
Recorded hyperuricaemia diagnosis	248/4,784	1.15 (1.02–1.30)	0.023
Diuretic users	548/5,771	1.23 (1.14–1.32)	<0.001
Sensitivity analyses			
Complete case	643/9,378	1.24 (1.16–1.33)	<0.001
Additionally adjusted for BP	615/7,118	1.21 (1.12–1.29)	<0.001
≥4 SUA measurements	569/7,542	1.22 (1.13–1.32)	<0.001
≥5 SUA measurements	504/5,541	1.20 (1.11–1.30)	<0.001
Daily aggregation; ≥3 distinct dates	624/10,274	1.22 (1.14–1.31)	<0.001
Additional ULT, colchicine and SGLT2i adjustment	649/10,556	1.25 (1.17–1.34)	<0.001
Restricted to eGFR <90	516/7,265	1.32 (1.22–1.43)	<0.001
Excluding NSAID users	168/3,033	1.35 (1.19–1.53)	<0.001
Exposure = SD of SUA	649/10,556	1.19 (1.12–1.28)	<0.001
Winsorised CV (1st-99th percentile)	649/10,556	1.25 (1.16–1.34)	<0.001
Post-90-day landmark (>90 d follow-up)	154/6,784	1.02 (0.86–1.21)	0.807

Model 3 adjustment as in Table 2; the defining covariate was omitted within the corresponding subgroup. The medication sensitivity additionally adjusted for any baseline urate-lowering therapy (ULT), colchicine and sodium-glucose cotransporter-2 inhibitor (SGLT2i) use. The post-90-day landmark retained participants with more than 90 days of follow-up and reset the time origin to day 90. BP, blood pressure; NSAID, non-steroidal anti-inflammatory drug.

### Secondary outcomes

The main composite kidney endpoint occurred in 676 participants (6.4%); the median time to the event was 283 days (IQR 150–409). Although the unadjusted 2-year curves separated across tertiles (Figure 2B), the adjusted association was null (HR per 1-SD CV 1.01, 95% CI 0.93–1.10). When a subsequent low eGFR was required after at least 28 days, 284 kidney events remained and the adjusted HR was 0.89 (95% CI 0.78–1.03); with a 90-day confirmation interval, 217 events remained and the HR was 0.89 (95% CI 0.76–1.04; Table 4). MACE occurred in 3,172 participants (30.0%). These were first composite events rather than recurrent episodes. The median time to MACE was 6 days (IQR 3–63); 1,754 (55.3%) occurred within 7 days and 2,452 (77.3%) within 90 days. The high incidence therefore reflected the hospital-based population, substantial baseline cardiovascular burden (coronary heart disease 59.6%, heart failure 38.2% and prior stroke 46.1%), a broad composite that included heart-failure hospitalisation, and marked early event clustering. Unadjusted curves showed modest separation (Figure 2C), whereas the adjusted association between CV and MACE was null (HR per 1-SD 1.02, 95% CI 0.98–1.05; Table 4).

**Table 4:** Secondary outcomes by serum uric acid variability.

Outcome/variability	Model 1	Model 2	Model 3 (full)
Main composite kidney endpoint (676 events)			
Per 1-SD increase in CV	1.17 (1.09–1.26)	1.16 (1.07–1.25)	1.01 (0.93–1.10)
Tertile 1	1.00	1.00	1.00
Tertile 2	1.37 (1.14–1.65)	1.39 (1.16–1.68)	1.15 (0.95–1.39)
Tertile 3	1.45 (1.20–1.76)	1.41 (1.17–1.71)	1.01 (0.82–1.24)
*p* for trend	<0.001	<0.001	0.979
MACE (3,172 first events)			
Per 1-SD increase in CV	1.06 (1.02–1.10)	1.02 (0.98–1.05)	1.02 (0.98–1.05)
Tertile 3 vs. 1	1.14 (1.04–1.24)	1.06 (0.97–1.15)	1.05 (0.96–1.16)
*p* for trend	0.004	0.202	0.241
Kidney-outcome definition sensitivities			
40% eGFR decline confirmed after ≥28 d (284 events)	–	–	0.89 (0.78–1.03)
40% eGFR decline confirmed after ≥90 d (217 events)	–	–	0.89 (0.76–1.04)

Values are HR (95% CI). The main kidney composite comprised kidney failure (maintenance dialysis, transplantation or eGFR <15 mL/min/1.73 m^2^) or the first observed ≥40% eGFR decline; confirmation was not required and transient acute kidney injury was not fully excluded. Sensitivity definitions required a subsequent eGFR remaining ≥40% below baseline after the stated interval. MACE denotes the first post-index qualifying event; recurrent events were not counted. Adjustment as in Table 2. Cox models used all available follow-up; the 2-year limit applies only to Figure 2. eGFR, estimated glomerular filtration rate; MACE, major adverse cardiovascular events.

### Subgroups and sensitivity analyses

Associations with mortality were observed across several high-risk subpopulations and were stronger among patients with eGFR <60 mL/min/1.73 m^2^ than among those with higher eGFR (HR 1.34 versus 1.09; *p* for interaction = 0.02; Figure 4). Estimates were similar in complete cases, after additional adjustment for blood pressure, when requiring at least four or five SUA measurements, and after winsorisation (Table 3; Figure 5). After averaging same-day SUA values and requiring at least three distinct measurement dates, 10,274 participants (624 deaths) remained and the HR was 1.22 (95% CI 1.14–1.31). Additional adjustment for baseline urate-lowering therapy, colchicine and SGLT2-inhibitor exposure produced an HR of 1.25 (95% CI 1.17–1.34; *p* < 0.001). Exploratory analyses in very elderly participants and in patients with or without cancer are reported in Supplementary Table S1 and Supplementary Figure S1. These analyses reduce but do not remove confounding by treatment changes or testing intensity.

**Figure 4:**
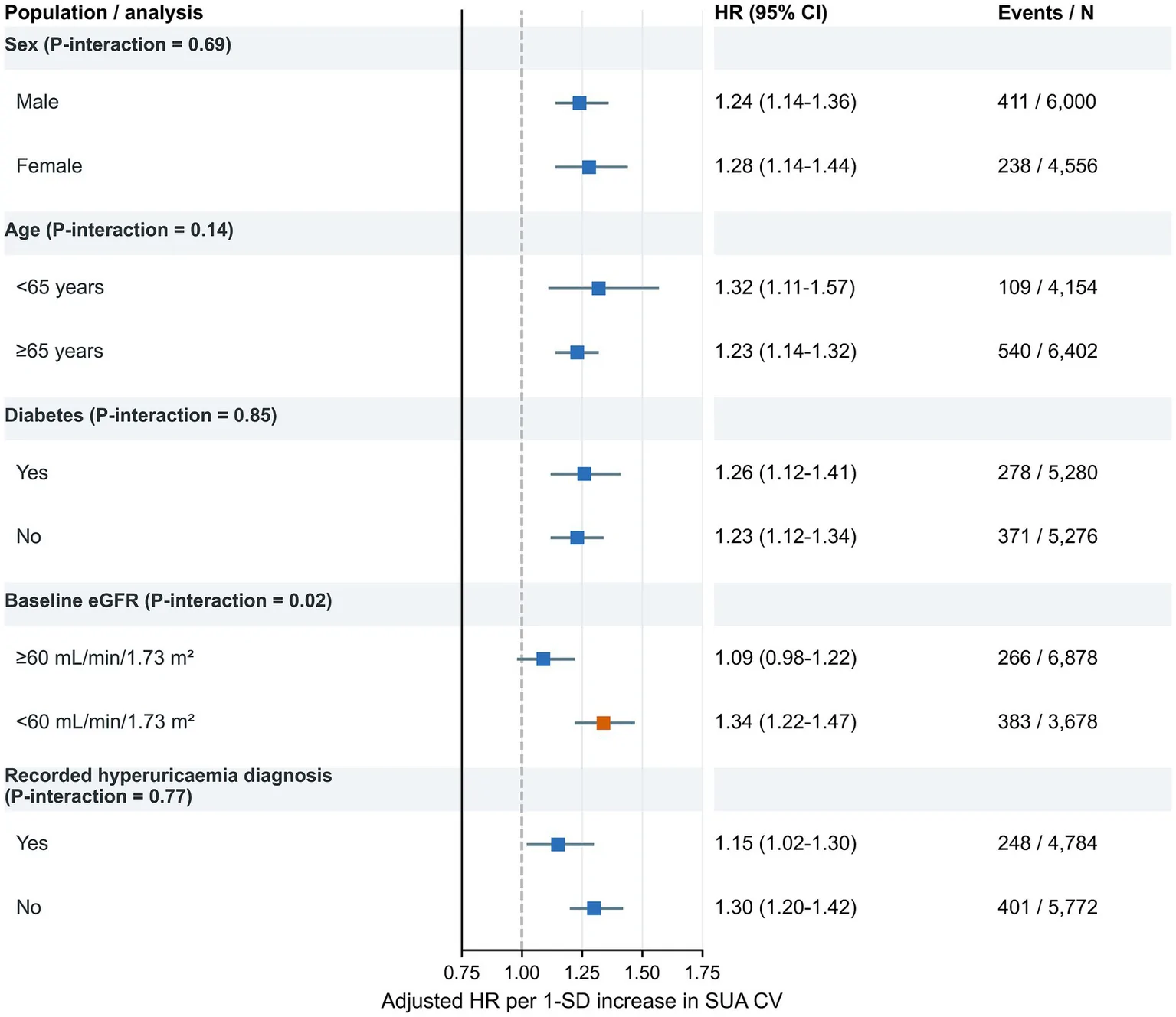
Subgroup analysis for all-cause mortality, expressed as fully adjusted hazard ratios per 1-SD increase in serum uric acid CV with interaction *p* values. Hyperuricaemia denotes a recorded diagnosis rather than a study-defined biochemical threshold.

**Figure 5:**
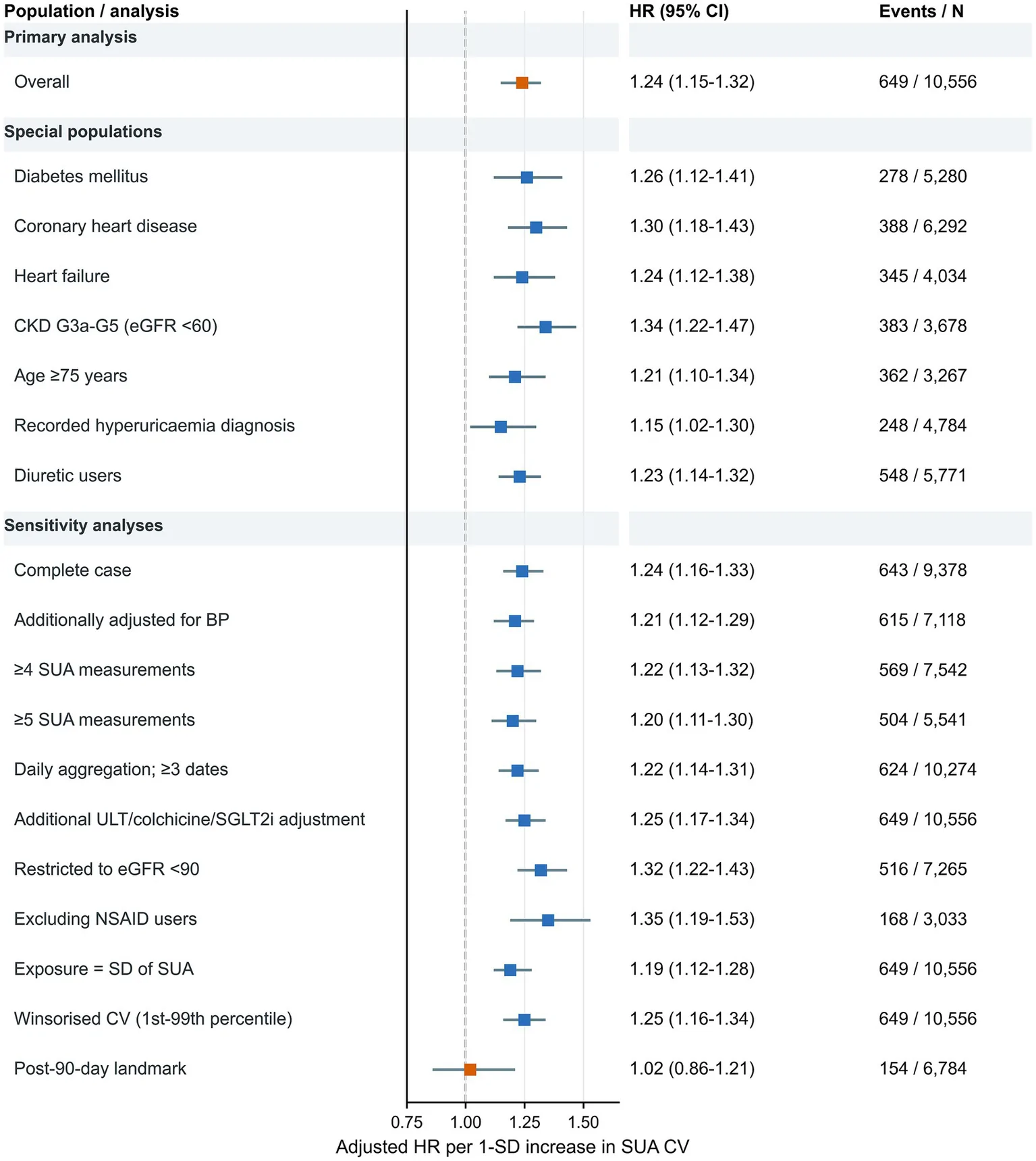
Special-population and sensitivity analyses for all-cause mortality, expressed as fully adjusted hazard ratios per 1-SD increase in serum uric acid CV. Full sensitivity results are reported in Table 3.

### Independent replication in MIMIC-IV

The MIMIC-IV analysis included 5,350 critically ill adults with at least three SUA measurements, among whom 931 died within 90 days. The CV distribution was similar to that of the Wuhan cohort (median 18.1% versus 18.5%). The adjusted HR per 1-SD increase in CV was 1.32 (95% CI 1.26–1.38), and the HR for the highest versus lowest tertile was 2.22 (95% CI 1.85–2.67; Table 5; Figure 6). This result demonstrates reproducibility of the short-term association in a critical-care population; it does not establish generalisability to ambulatory or population-based CKD cohorts.

**Table 5:** Independent replication in MIMIC-IV: Serum uric acid variability and 90-day all-cause mortality (*n* = 5,350).

Exposure	Events/*n*	HR (95% CI)	*p*
Per 1-SD CV (crude)	931/5,350	1.48 (1.43–1.54)	<0.001
Per 1-SD CV (adjusted)	931/5,350	1.32 (1.26–1.38)	<0.001
Tertile 2 vs. 1 (adjusted)	245/1,783	1.30 (1.07–1.58)	0.009
Tertile 3 vs. 1 (adjusted)	514/1,784	2.22 (1.85–2.67)	<0.001
Per 1-SD CV, eGFR <60	442/1,641	1.31 (1.22–1.40)	<0.001

Adjusted for age, sex, mean SUA, number of measurements and eGFR (2021 CKD-EPI equation). For tertile contrasts, Events/*n* refer to the listed tertile; tertile 1 is the reference. MIMIC-IV is a critical-care population; replication supports reproducibility of the short-term association but not generalisability to other ambulatory CKD cohorts. CV, coefficient of variation; eGFR, estimated glomerular filtration rate.

**Figure 6:**
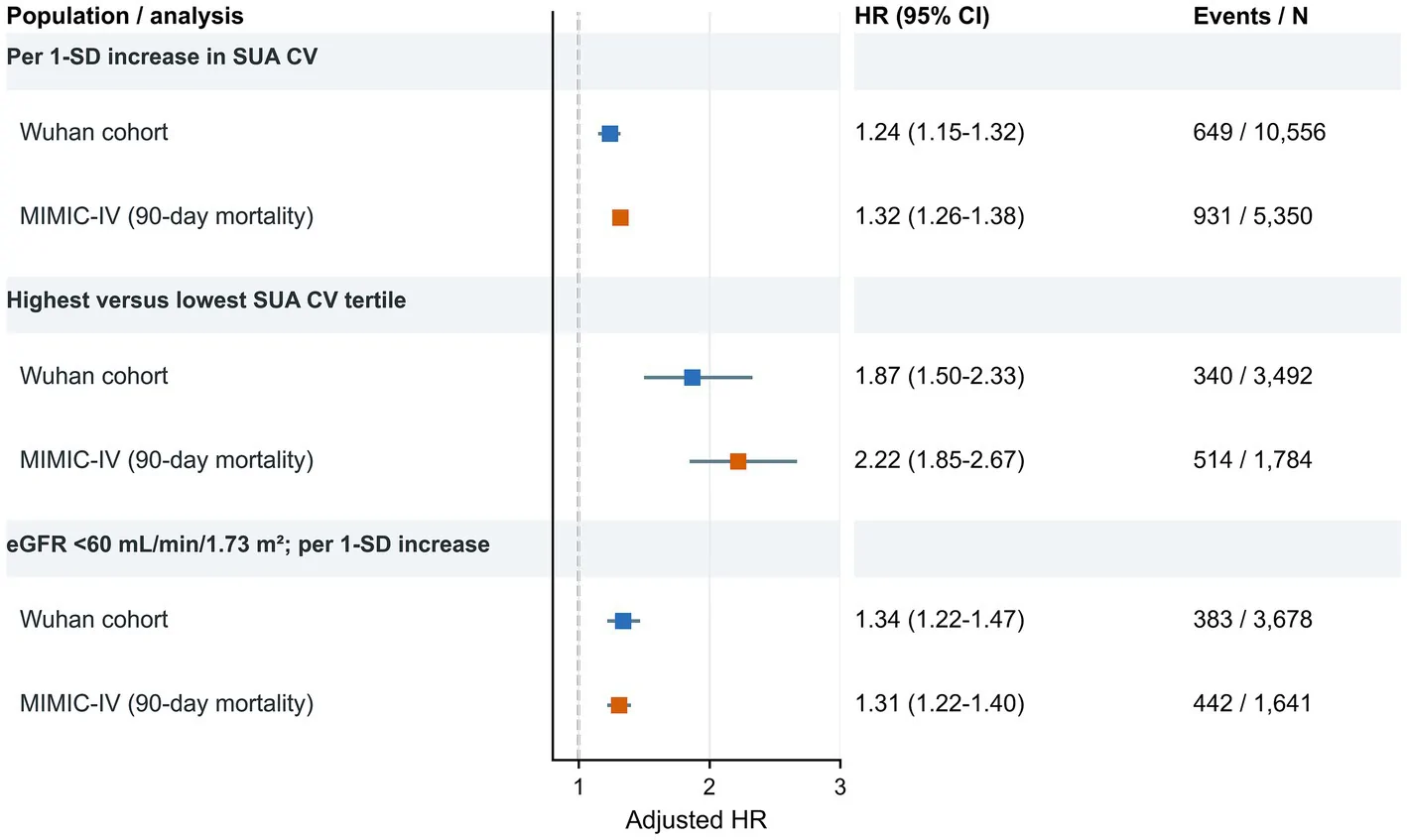
Comparison of fully adjusted mortality hazard ratios in the Wuhan cohort and the independent MIMIC-IV replication cohort. For highest-versus-lowest-tertile contrasts, Events/*n* refer to the highest tertile; tertile 1 is the reference. The Wuhan estimate concerns mortality during the observed short follow-up; MIMIC-IV estimates concern 90-day mortality in critically ill adults. Concordance supports reproducibility of a short-term association, not transportability to another ambulatory CKD cohort.

## Discussion

In this nine-hospital cohort of adults with EHR-defined CKD, greater variability in serial SUA measurements was associated with all-cause mortality after multivariable adjustment. The association was robust to alternative variability measures, same-day aggregation, stricter measurement requirements and additional adjustment for medications that directly or indirectly affect SUA. However, three observations materially constrain interpretation: median follow-up was only 0.62 years, more than three quarters of deaths occurred within 90 days, and an association was not observed after the 90-day landmark. SUA variability should therefore be regarded primarily as a marker of near-term clinical instability, not as evidence of a causal mechanism or a durable long-term prognostic factor.

Earlier studies associated SUA variability with incident cardiovascular events in type 2 diabetes (20) and with coronary heart disease and all-cause mortality in a working population (21), while time-updated eGFR variability has been associated with mortality and cardiorenal outcomes in CKD (22). Our results extend this variability literature to a hospital-based CKD cohort but also show that timing is critical: deaths were concentrated early, and an association was not detected in the post-90-day landmark analysis.

The temporal pattern is compatible with reverse causation. Acute illness, decompensated heart or kidney disease, changes in volume status, inflammation, reduced intake, treatment changes and end-of-life care can all alter urate production or renal handling while simultaneously increasing near-term mortality. A high CV calculated from routine-care measurements may summarise this instability. This marker-not-mechanism interpretation is consistent with CKD-FIX and PERL, in which allopurinol-mediated urate lowering did not slow eGFR loss (23, 24), and with trial evidence on the cardiovascular safety rather than cardiovascular benefit of urate-lowering therapy (25). Although xanthine oxidoreductase links urate metabolism to oxidative stress (26–28), the present data cannot distinguish a biological effect of fluctuation from illness-induced fluctuation.

The requirement for at least three measurements creates an important selection mechanism. Patients who undergo repeated testing may have more severe CKD, gout, unstable metabolic status, medication changes, greater healthcare use or intercurrent illness. Adjustment for measurement number and similar findings after requiring four or five measurements do not eliminate this bias. The median interval between distinct dates was 26 days but varied widely, and nearly one quarter of participants had same-day duplicates. Daily aggregation produced a similar estimate, yet unequal spacing and clinically triggered testing remain inseparable from the exposure. Residual selection and surveillance bias are therefore likely.

Medication use is another potential source of variability. Urate-lowering agents, diuretics, NSAIDs, RAAS inhibitors and SGLT2 inhibitors can change SUA concentrations, and treatment may start, stop or change during clinical deterioration. SGLT2 inhibitors reduce SUA and incident gout in observational and trial-related analyses (29, 30), and longitudinal changes in SUA have been associated with mortality (31), but these findings do not show that reducing variability itself improves outcomes. The association in our cohort was essentially unchanged after adjustment for urate-lowering therapy, colchicine and SGLT2 inhibitors, but prescription data do not establish adherence and time-updated dose changes were not modelled. The sensitivity analysis therefore addresses measured baseline exposure only and cannot exclude treatment-confounding or indication bias.

The null adjusted associations with kidney progression and MACE also require careful interpretation. The original kidney composite used the first observed 40% eGFR decrease, which could capture transient acute kidney injury; requiring confirmation after 28 or 90 days reduced event counts substantially and did not reveal an association. The high MACE incidence reflected a broad first-event composite, substantial prevalent cardiovascular disease and very early hospital-based event ascertainment rather than recurrent-event counting. Because death was common early and the follow-up window was short, longer-term non-fatal kidney and cardiovascular outcomes may also have been under-ascertained.

The MIMIC-IV result strengthens evidence that the short-term association is reproducible when SUA is repeatedly measured during serious illness. It should not be described as validation in a comparable ambulatory CKD cohort. MIMIC-IV patients are critically ill, measurements are often closely spaced, covariate adjustment was more limited, and the outcome was deliberately restricted to 90-day mortality. Concordant estimates across these distinct EHR settings support a common near-term instability signal, but not transportability to stable community-dwelling patients with CKD.

Strengths include the large multicentre sample, multiple variability metrics, explicit analysis of measurement timing, several medication and exposure-definition sensitivities, evaluation of three clinical outcomes, and independent replication in MIMIC-IV. Limitations remain substantial. The study was retrospective, hospital-based and confined to nine institutions in one city. CKD was defined through a pragmatic EHR phenotype; chronicity of at least 3 months was not verified for every participant, albuminuria was incompletely available, and transient dysfunction or coding error may have been misclassified. Requiring repeated SUA testing may select clinically sicker, more frequently observed patients. Measurement intervals were irregular and care setting could not be reliably distinguished. Outcome ascertainment was algorithmic rather than manually adjudicated, the unconfirmed main kidney definition could include acute kidney injury, and prescription records could not establish adherence. Median follow-up was short; the global proportional-hazards test was significant although the exposure-specific test was not. Residual confounding and reverse causation are likely. Prospective cohorts with protocolised serial SUA measurements, longer follow-up and time-updated clinical data are needed before SUA variability is used for long-term risk prediction or intervention decisions.

In conclusion, greater variability in serial SUA measurements was associated with mortality during short follow-up in adults with EHR-defined CKD and the short-term association was reproduced in MIMIC-IV. Because deaths were concentrated within 90 days and an association was not observed in the post-90-day landmark analysis, SUA variability may be a marker of near-term clinical deterioration rather than evidence of a causal or durable long-term prognostic mechanism. Adjusted associations with kidney progression and MACE were null.

## Data Availability

The raw data supporting the conclusions of this article will be made available by the authors, without undue reservation.
