# Supplementary material for: Serum uric acid variability and short-term mortality in chronic kidney disease: a retrospective cohort study with independent replication in MIMIC-IV
Source: Front Nutr. 2026 Sep 16;13:1919779. doi: 10.3389/fnut.2026.1919779 (PMC13623910; doi:10.3389/fnut.2026.1919779)
Supplement: Supplementary file 1 [file Data_Sheet_1.docx]

STROBE Checklist for Cohort Studies

Completed reporting checklist | Manuscript ID 1919779

**Manuscript title:** Serum uric acid variability and short-term mortality in chronic kidney disease: a retrospective cohort study with independent replication in MIMIC-IV

**Checklist basis:** STROBE Statement—checklist of items that should be included in reports of cohort studies. Official checklist: https://www.strobe-statement.org/checklists/

**Location convention: Items marked “Not reported” or “Not applicable” are disclosed transparently rather than being inferred.**

**COMPLETION STATUS**

All 22 STROBE items and cohort-specific sub-items are mapped below. The checklist is a reporting aid and does not imply that every optional item is applicable or separately reported.

| **Section** | **Item** | **Recommendation** | **Location in article** | **Completion note** |
| --- | --- | --- | --- | --- |
| **Title and abstract** | **1a** | Indicate the study design with a commonly used term in the title or abstract. | Title; Abstract | Retrospective cohort study identified in the title and abstract. |
|  | **1b** | Provide an informative and balanced abstract summary of what was done and found. | Abstract | Includes design, cohorts, exposure, outcomes, adjusted estimates, landmark result, replication and interpretation. |
| **Introduction** | **2** | Explain the scientific background and rationale. | Introduction | Clinical background, biomarker-variability rationale and uncertainty are described. |
|  | **3** | State specific objectives, including prespecified hypotheses. | Introduction | Objectives, time-horizon focus and non-causal estimand are stated. |
| **Methods** | **4** | Present key elements of the study design early in the paper. | Materials and methods—Study design and population | Retrospective EHR cohort, eligibility, index date and cohort construction are stated. |
|  | **5** | Describe setting, locations and relevant dates/periods, including recruitment, exposure, follow-up and data collection. | Materials and methods—Study design and population; SUA exposure and measurement timing; Outcomes and ascertainment; Independent replication in MIMIC-IV | Nine-hospital Wuhan EHR setting, 365-day exposure window, index date, follow-up mechanism and MIMIC-IV setting are reported. Calendar study dates are not separately reported. |
|  | **6a** | Give eligibility criteria and the sources and methods of participant selection and follow-up. | Materials and methods—Study design and population; Outcomes and ascertainment | Eligibility, exclusions, data source and follow-up are reported. |
|  | **6b** | For matched studies, give matching criteria and numbers of exposed and unexposed participants. | Not applicable—the cohort was not matched | The cohort was not matched. |
|  | **7** | Clearly define outcomes, exposures, predictors, confounders and effect modifiers; give diagnostic criteria when applicable. | Materials and methods—Study design and population; SUA exposure and measurement timing; Outcomes and ascertainment; Covariates and medication exposure | SUA variability, CKD, mortality, kidney outcome, MACE, covariates and medication exposure are defined. |
|  | **8** | For each variable, give data sources and assessment methods; describe comparability across groups if relevant. | Materials and methods—SUA exposure and measurement timing; Outcomes and ascertainment; Independent replication in MIMIC-IV | Structured EHR sources, equations, outcome algorithms and the MIMIC-IV comparison boundary are stated. |
|  | **9** | Describe efforts to address potential sources of bias. | Materials and methods—Statistical analysis; Discussion | Adjustment, timing, landmark and sensitivity analyses are reported; residual reverse causation, selection, surveillance and misclassification are acknowledged. |
|  | **10** | Explain how the study size was determined. | Materials and methods—Study design and population; Figure 1 | All eligible records meeting the prespecified criteria were included; no formal a priori sample-size calculation was performed. |
|  | **11** | Explain how quantitative variables were handled; describe groupings and why they were chosen. | Materials and methods—SUA exposure and measurement timing; Statistical analysis | CV, ASV, VIM, 1-SD scaling, tertiles and restricted cubic splines are described. |
|  | **12a** | Describe all statistical methods, including those used to control for confounding. | Materials and methods—Statistical analysis | Nested Cox models, covariate adjustment, splines, interactions, diagnostics and sensitivity analyses are described. |
|  | **12b** | Describe methods used to examine subgroups and interactions. | Materials and methods—Statistical analysis; Results—Subgroups and sensitivity analyses; Figure 4 | Prespecified subgroup models and interaction terms are reported. |
|  | **12c** | Explain how missing data were addressed. | Materials and methods—Missing data | Variable-specific missingness, single median replacement and complete-case sensitivity are reported. |
|  | **12d** | If applicable, explain how loss to follow-up was addressed. | Materials and methods—Outcomes and ascertainment | Follow-up ended at outcome, death or last recorded contact; no additional loss-to-follow-up model was used. |
|  | **12e** | Describe sensitivity analyses. | Materials and methods—Statistical analysis; Results—Timing of mortality and reverse-causation analysis; Secondary outcomes; Subgroups and sensitivity analyses; Tables 3–4 | Landmark, alternative exposure, complete-case, measurement, medication and kidney-confirmation sensitivities are reported. |
| **Results** | **13a** | Report numbers at each stage of the study. | Materials and methods—Study design and population; Figure 1 | Potentially eligible, excluded and included counts are reported and shown in Figure 1. |
|  | **13b** | Give reasons for non-participation at each stage. | Materials and methods—Study design and population; Figure 1 | Reasons include incomplete follow-up/missing index information and age under 18 years. |
|  | **13c** | Consider use of a flow diagram. | Figure 1 | A study flow diagram is provided. |
|  | **14a** | Give participant characteristics and information on exposures and potential confounders. | Results—Cohort characteristics and SUA measurement timing; Table 1 | Baseline characteristics and SUA measurement timing are reported. |
|  | **14b** | Indicate the number of participants with missing data for each variable of interest. | Materials and methods—Missing data | Counts and percentages are reported for albumin, haemoglobin and glucose; eGFR and mean SUA were complete. |
|  | **14c** | Summarise follow-up time. | Results—SUA variability and all-cause mortality | Median, IQR and total person-years are reported. |
|  | **15** | Report numbers of outcome events or summary measures over time. | Results—SUA variability and all-cause mortality; Timing of mortality and reverse-causation analysis; Secondary outcomes; Independent replication in MIMIC-IV | Death, kidney and first-MACE counts and timing are reported. |
|  | **16a** | Give unadjusted and adjusted estimates with precision; state which confounders were adjusted for and why. | Results—SUA variability and all-cause mortality; Secondary outcomes; Independent replication in MIMIC-IV; Tables 2, 4, and 5 | Unadjusted and adjusted HRs with 95% CIs are reported; model covariates are listed in the Table 2 note. |
|  | **16b** | Report category boundaries when continuous variables were categorised. | Materials and methods—SUA exposure and measurement timing; Table 2 | CV was grouped into cohort tertiles; tertile 1 is the reference. Numeric cutpoints are not separately tabulated. |
|  | **16c** | If relevant, translate relative risk estimates into absolute risk for a meaningful period. | Not separately reported—event counts, follow-up, and cumulative-incidence curves are provided in the Results and Figure 2 | The study reports event counts, follow-up and cumulative-incidence curves; no separate absolute-risk translation was performed. |
|  | **17** | Report other analyses, such as subgroup, interaction and sensitivity analyses. | Results—Subgroups and sensitivity analyses; Independent replication in MIMIC-IV; Tables 3–5; Figures 4–6 | Subgroups, multiple sensitivities and MIMIC-IV replication are reported. |
| **Discussion** | **18** | Summarise key results with reference to study objectives. | Discussion | The main mortality result and its temporal constraints are summarised. |
|  | **19** | Discuss limitations, including potential bias or imprecision, and their direction and magnitude. | Discussion | Reverse causation, testing intensity, selection/surveillance bias, outcome misclassification, treatment confounding, CKD misclassification, short follow-up and transportability are discussed. |
|  | **20** | Give a cautious overall interpretation considering objectives, limitations, multiplicity and other evidence. | Discussion | Interpretation is explicitly associative, short-term and non-causal. |
|  | **21** | Discuss generalisability. | Results—Independent replication in MIMIC-IV; Discussion | MIMIC-IV supports critical-care reproducibility but not ambulatory/population CKD transportability. |
| **Other information** | **22** | Give the source of funding and the role of funders. | Funding | Funding source and grant number are reported; no funder role is stated. |
