# Supplementary material for: Serum uric acid variability and short-term mortality in chronic kidney disease: a retrospective cohort study with independent replication in MIMIC-IV
Source: Front Nutr. 2026 Sep 16;13:1919779. doi: 10.3389/fnut.2026.1919779 (PMC13623910; doi:10.3389/fnut.2026.1919779)
Supplement: Supplementary file 2 [file Supplementary_file_1.DOCX]

**Supplementary Material**

Manuscript ID 1919779

*Serum uric acid variability and short-term mortality in chronic kidney disease: a retrospective cohort study with independent replication in MIMIC-IV*

# Supplementary Table S1

Exploratory special-population analyses for all-cause mortality according to continuous SUA variability

| **Population** | **Deaths / N** | **Adjusted HR per SD increase in SUA CV (95% CI)** | **P value** |
| --- | --- | --- | --- |
| **Age ≥80 years** | 273 / 2,148 | 1.25 (1.12–1.40) | <0.001 |
| **Age ≥85 years** | 169 / 1,097 | 1.18 (1.02–1.37) | 0.028 |
| **Age ≥90 years** | 71 / 325 | 1.19 (0.96–1.47) | 0.104 |
| **Any recorded cancer** | 316 / 2,083 | 1.19 (1.07–1.31) | <0.001 |
| **Recorded solid tumour** | 303 / 1,968 | 1.22 (1.10–1.35) | <0.001 |
| **Recorded haematological malignancy** | 30 / 182 | 1.16 (0.76–1.77) | 0.491 |
| **No recorded cancer** | 333 / 8,473 | 1.27 (1.15–1.41) | <0.001 |

*Values are hazard ratios (HRs) from the fully adjusted Cox model (Model 3). These analyses were exploratory and were not adjusted for multiplicity. CV, coefficient of variation; SUA, serum uric acid.*

# Supplementary Figure S1

Figure S1. Exploratory adjusted associations between serum uric acid variability and all-cause mortality in selected older and cancer-defined populations. Squares represent adjusted hazard ratios per 1-standard-deviation increase in SUA coefficient of variation; horizontal lines represent 95% confidence intervals. The vertical line denotes a hazard ratio of 1. Estimates are from the fully adjusted Model 3 and were not adjusted for multiplicity.
